# Influence of Oil Shale Residue on the Frost Resistance of Fly-Ash-Based Autoclaved Aerated Concrete

**DOI:** 10.3390/ma19173675

**Published:** 2026-08-29

**Authors:** Lei Zhao, Yao Xiao, Jiayu Luo, Wenqi E, Xinze Gao, Cong Zeng

**Affiliations:** 1 Economic and Technical Research Institute, State Grid Heilongjiang Electric Power Co., Ltd., Harbin 150090, China; zhaolei19870212@126.com (L.Z.); zoey93415/@163.com (Y.X.); shaw6770@foxmail.com (J.L.); 2 School of Civil Engineering, Northeast Electric Power University, 169 Changchun Road, Jilin 132012, China; 2202400959@neepu.edu.cn (X.G.); zc_1113@126.com (C.Z.)

**Keywords:** autoclaved aerated concrete, fly ash, oil shale residue, frost resistance, phase change material, solid waste utilization

## Abstract

To promote the high-value utilization of industrial solid waste and enhance the durability of construction materials in cold regions, this study investigates an autoclaved aerated concrete (AAC) system prepared primarily with fly ash and oil shale residue, with a particular focus on its frost resistance. The physical and chemical properties of the raw materials were characterized using X-ray fluorescence (XRF) and X-ray diffraction (XRD) to optimize the pore structure and mineral composition. A systematic evaluation of 15 continuous freeze–thaw cycles was conducted, comprehensively analyzing the compressive strength retention, mass loss rate, and thermal conductivity. The experimental results indicate that a 50% replacement of fly ash with oil shale residue, combined with an optimized water-to-binder ratio (0.66), significantly improves the pore uniformity and skeleton stability of the AAC. The optimized mixture (YYY50W) achieved a compressive strength of 5.2 MPa and a low thermal conductivity of 0.1654 W/(m·K). After 15 freeze–thaw cycles, the mass loss was minimal (<3 g), and the compressive strength retention rate ($R$) reached 90.0%, demonstrating superior frost resistance. This study elucidates the microstructural mechanism by which oil shale residue promotes the formation of low-crystallinity tobermorite and C-S-H gels, providing potential experimental evidence for the utilization of industrial by-products in building materials for cold environments.

## 1. Introduction

Autoclaved aerated concrete (AAC) is a lightweight and porous building material, but its application in cold regions is often restricted by frost-induced microcracking and structural degradation [1,2,3,4]. Fundamentally, the frost damage in AAC is governed by the liquid-to-solid phase transition of water within its complex pore network. During freeze–thaw cycles, the volumetric expansion of freezing water generates severe hydrostatic and crystallization pressures. If these internal stresses exceed the tensile capacity of the pore walls, macroscopic spalling and drastic strength deterioration occur [5,6]. Therefore, tailoring the internal pore architecture—specifically, maximizing the disconnected, closed-pore ratio to act as expansion buffers—has become a consensus for improving the frost durability of lightweight concrete [7].

Recently, recycling industrial solid wastes like fly ash and oil shale residue into AAC has emerged as a promising strategy to reduce carbon footprints and raw material costs [8,9,10,11]. Specifically, oil shale residue contains abundant reactive silica and alumina, which facilitate the formation of hydrothermal products such as tobermorite and calcium silicate hydrate (C-S-H) gels [12].

Recent investigations have extensively explored introducing diverse industrial by-products into AAC to modify its microstructure. For instance, recent studies demonstrated that incorporating specific industrial tailings and coal gangue could refine the pore distribution and enhance mechanical retention post-freezing, though often at the cost of compromised thermal insulation [13,14]. Similarly, while pure fly-ash-based AAC offers excellent initial insulation, its highly interconnected capillary pores remain vulnerable to rapid water ingress and subsequent frost heave [15]. Compared to these conventional additives, as highlighted in a recent systematic review [16], oil shale residue acts as an excellent supplementary cementitious material due to its dual self-cementing and pozzolanic properties.

Consequently, researchers have successfully explored its high-volume application in mineral foam blocks [17] and sustainable mortars [18], demonstrating that optimized mix proportions can significantly refine micro-porosity. Furthermore, studies on AAC incorporating other solid wastes confirm that optimizing the micro-pore network is fundamental to suppressing thermal conductivity and enhancing frost resistance [19,20,21]. Despite these advancements, the high-volume utilization of oil shale residue in AAC remains a challenge. Its high water absorption and irregular particle morphology often cause rapid slurry thickening and mismatched gas-generation rates, ultimately deteriorating the pore structure and limiting its replacement ratio [22,23]. Consequently, the microstructural evolution and frost-resistance mechanisms of AAC containing high volumes of oil shale residue under cyclic freeze–thaw conditions remain poorly understood.

To address this critical gap, this study develops a high-performance AAC system utilizing a large volume of oil shale residue as a partial replacement for fly ash. By optimizing the water-to-binder ratio, the adverse effects on slurry flowability were effectively mitigated. A comprehensive experimental program—including steady-state thermal conductivity measurements and 15 continuous freeze–thaw cycles—was conducted. Furthermore, microstructural analyses via mercury intrusion porosimetry (MIP), X-ray Diffraction (XRD), and Scanning Electron Microscopy (SEM) were employed to elucidate the hydration behavior and pore structure evolution. By systematically comparing our macroscopic and microstructural results with contemporary studies on traditional fly ash and other solid-waste-based AACs, these findings are expected to provide a robust experimental and theoretical basis for enhancing the durability of solid-waste-based AAC in cold climates.

## 2. Materials and Methods

### 2.1. Raw Materials

The raw materials used in this study primarily consisted of Portland cement, fly ash, oil shale residue, quicklime, gypsum, and aluminum powder. P.O 42.5 ordinary Portland cement (Yatai Cement Co., Ltd., Jilin, China) was used as the primary calcareous material to provide early strength. Fly ash served as the standard siliceous material, while the oil shale residue was obtained from an industrial refinery plant located in Fushun, China. To ensure adequate reactivity and proper particle grading, the crude oil shale residue was ground using a horizontal ball mill to pass through a 2.36 mm standard sieve prior to use. Additionally, locally sourced quicklime with an active CaO content exceeding 80% was utilized as the alkaline activator. Gypsum was added to regulate the hydration rate, and aluminum powder with a fineness passing through a 0.088 mm sieve (Xinfa Aluminum Co., Ltd., Liaocheng, China) acted as the gas-generating agent.

**Figure 1:**
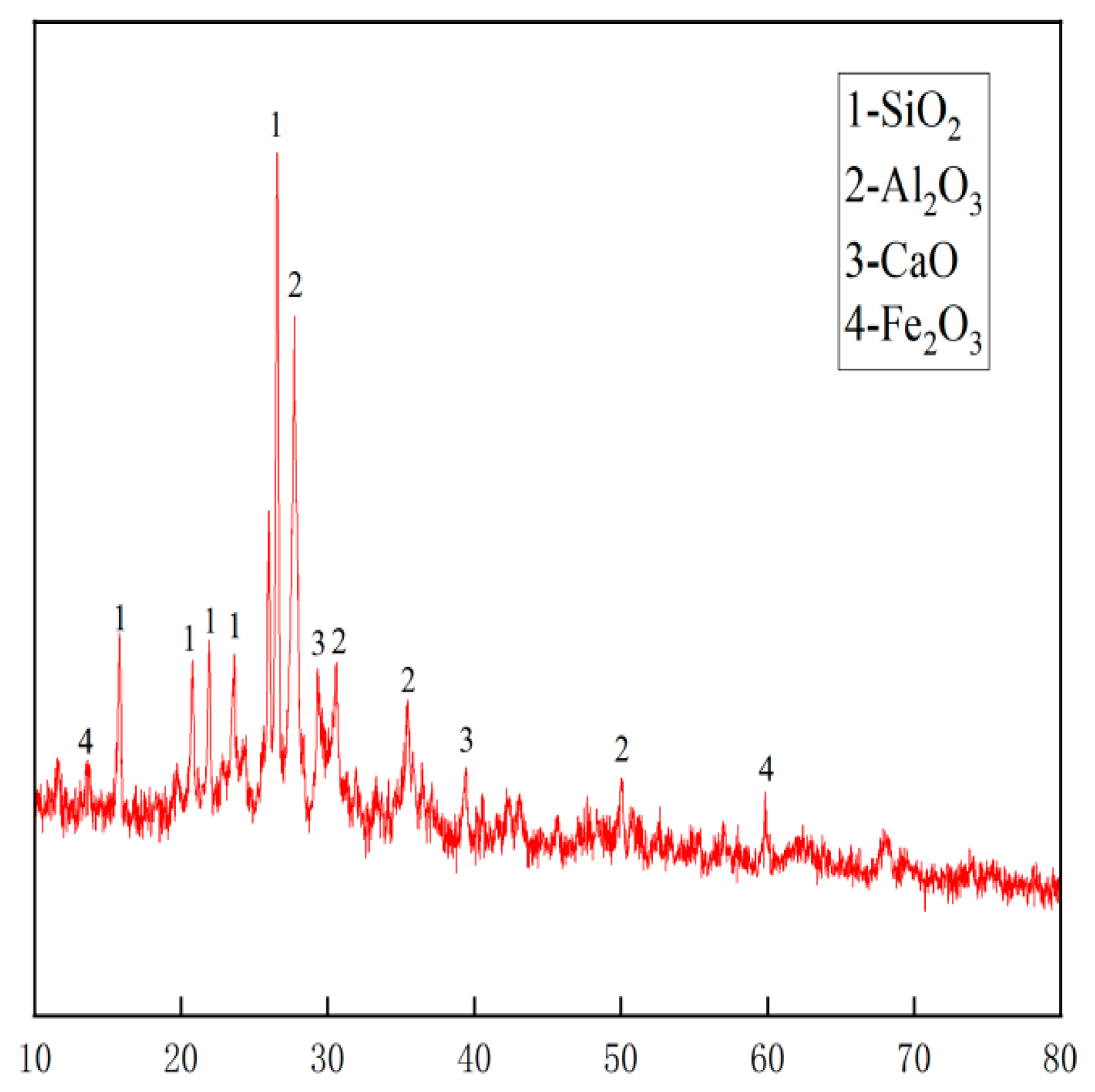
X-ray diffraction (XRD) pattern of oil shale residue, showing the identified crystalline mineral phases, mainly quartz, feldspar, calcite, and dehydrated clay minerals.

The chemical compositions of the primary solid materials were determined using X-ray fluorescence (XRF), and the results are summarized in Table 1. The XRF results show that the oil shale residue is mainly composed of SiO_2_, Al_2_O_3_, CaO, and Fe_2_O_3_. It should be noted that these oxides are mainly introduced by mineral phases such as quartz, feldspar, calcite, and dehydrated clay minerals, as identified in the XRD pattern of the raw material (Figure 1).

### 2.2. Mix Proportion Design

To investigate the effect of oil shale residue on AAC and optimize the performance, five distinct mixtures were designed. A baseline mixture (SN20) was formulated using standard proportions. As illustrated in Table 2, the ingredients are logically categorized into siliceous materials (which are subjected to substitution) and calcareous materials (which remain constant). Oil shale residue was introduced to replace fly ash at 50% and 100% by mass. Based on preliminary trials, introducing high amounts of oil shale residue significantly reduced slurry flowability. Therefore, modified groups (YYY50W and YYY100W) were designed by adjusting the water-to-binder (W/B) ratio from 0.60 to 0.66 and increasing the aluminum powder dosage from 0.090% to 0.095% to maintain pore stability and optimal density. The detailed mixture proportions are presented in Table 2.

### 2.3. Specimen Preparation and Autoclaving

The dry powders (fly ash, oil shale residue, and gypsum) were pre-mixed for 60 s with warm water (50 °C). Lime was then added and mixed for another 60 s, followed by cement addition and mixing for 120 s. Finally, the pre-suspended aluminum powder was poured into the slurry and mixed for 30 s. The mixture was cast into 100 mm × 100 mm × 100 mm molds and pre-cured at 50 °C for 2 h. After the top expansion was removed, the specimens were demolded and transferred to an autoclave, where they were cured under a saturated steam pressure of 1.4 MPa at 180 °C for 8 h.

### 2.4. Testing Methods

All experimental tests were conducted with three independent replicates per group (*n* = 3 for each specific test), and the results were expressed as mean values ± standard deviations (SDs) to ensure statistical reliability. All data analysis and graphical visualizations were processed using OriginPro 2021 software (OriginLab Corporation, Northampton, MA, USA).

Compressive strength and absolute dry density were tested in accordance with the GB/T 11969-2020 standard [20]. Prior to testing, the samples were dried at 105 °C to a constant mass. The thermal conductivity of the specimens was measured using a DRPL-III steady-state thermal conductivity analyzer (Xiangtan Instrument Co., Ltd., Xiangtan, China) strictly in accordance with the ISO 8301 standard [21], based on one-dimensional steady heat transfer principles.

The freeze–thaw resistance was evaluated according to the GB/T 11969-2020 standard [20]. Prior to the test, the specimens were preconditioned by complete immersion in water at 20 ± 2 °C for 48 h to achieve full saturation. A total of 15 fully saturated specimens (3 per group) were then subjected to 15 continuous rapid freeze–thaw cycles. Each cycle consisted of freezing in air at −15 ± 2 °C for at least 8 h, followed by thawing in a water bath at 20 ± 2 °C for at least 6 h. Visual inspections were conducted and recorded before and after the testing regime to monitor any macroscopic damage (e.g., surface spalling or cracking). After completing the 15 cycles, the mass loss rate and the compressive strength retention rate were calculated.

For microstructural analysis, hydration products were characterized utilizing a Bruker D8 Advance X-ray diffractometer (XRD, Karlsruhe, Germany) equipped with Cu Kα radiation. To accurately distinguish between open and closed pores, the pore parameters (e.g., average pore diameter and closed-pore ratio) were quantified using mercury intrusion porosimetry (MIP). Furthermore, the micro-matrix of the AAC specimens was observed using an S-4800 Scanning Electron Microscope (SEM, Hitachi, Tokyo, Japan).

## 3. Results and Discussion

### 3.1. Pore Structure Characteristics (MIP Analysis)

The macroscopic performance of AAC is inherently dependent on its internal pore characteristics. To scientifically validate the influence of oil shale residue on the pore network, the pore structure parameters obtained from mercury intrusion porosimetry (MIP) and image analysis are summarized in Table 3.

As shown in Table 3, the average pore diameter of the baseline group (SN20) was approximately 160.2 μm. When 50% oil shale residue was added without W/B optimization (YYY50), the high water absorption of the residue caused rapid slurry thickening [8,17]. This disrupted the gas-generation balance, resulting in a poor pore distribution and a sharp drop in the closed-pore ratio (to 55.1%). However, by optimizing the W/B ratio to 0.66 (YYY50W), the total porosity was maintained at 72.3%, the average pore diameter was refined to 95.8 μm, and crucially, the closed-pore ratio increased significantly to 78.6%. These robust MIP results mathematically confirm that the optimized water-to-binder ratio ensures uniform gas expansion and builds a highly disconnected (closed) pore network.

### 3.2. Thermal Conductivity and Mechanical Properties

**Figure 2:**
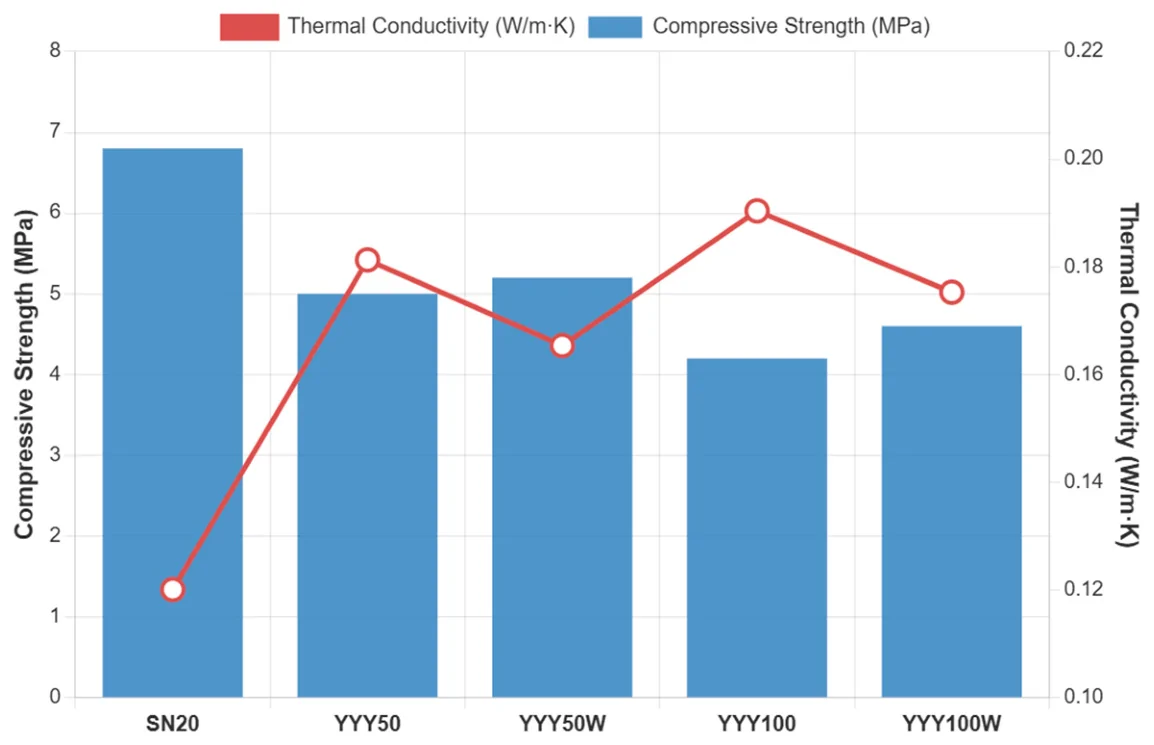
Physical and mechanical properties of AAC specimens.

The mechanical properties and dry density of the autoclaved specimens are summarized visually in Figure 2.

As clearly shown in Figure 2, the baseline SN20 exhibits the highest initial compressive strength (6.8 MPa) and the lowest thermal conductivity (0.1201 W/m·K). It is an undeniable fact that the direct addition of oil shale residue inherently introduces complex impurities and alters the hydration kinetics, exerting a slightly negative effect on the absolute initial macroscopic properties compared to the pure fly ash system. This initial strength reduction at high substitution levels is highly consistent with recent findings regarding oil shale ash application in cementitious mortars [12]. However, the primary objective of this study is to achieve the high-volume utilization of challenging solid waste through an acceptable performance trade-off. Despite the reduction, the optimized YYY50W group successfully achieved a compressive strength of 5.2 ± 0.2 MPa and an absolute dry density of 730 ± 11 kg/m^3^, fully satisfying the Chinese national standard (GB/T 11968-2020) [22] for structural Grade A5.0 B07 AAC blocks.

Furthermore, thermal conductivity is heavily dependent on pore structure connectivity [23]. When oil shale was un-optimized (YYY50), the thermal conductivity spiked to 0.1813 W/m·K due to the low closed-pore ratio (55.1%) reported in Table 3, which facilitated heat transfer via interconnected air convection. Remarkably, by utilizing the optimized mix (YYY50W), the thermal conductivity was successfully suppressed to 0.1654 ± 0.006 W/m·K. The significant increase in the closed-pore ratio (78.6%) effectively trapped stagnant air within the matrix, keeping the thermal insulation well within practical application limits [9].

This thermal-mechanical balance is particularly notable when compared to contemporary research. For instance, recent studies utilizing other complex solid wastes (such as coal gangue or iron tailings) in AAC often report thermal conductivities exceeding 0.18 W/(m·K) at comparable density levels [13,14], primarily due to a high proportion of interconnected capillary pores. In stark contrast, the 78.6% closed-pore network achieved in our optimized YYY50W matrix effectively interrupts continuous heat transfer pathways, providing a significantly more energy-efficient solution for structural insulation.

### 3.3. Thermal Conductivity

**Figure 3:**
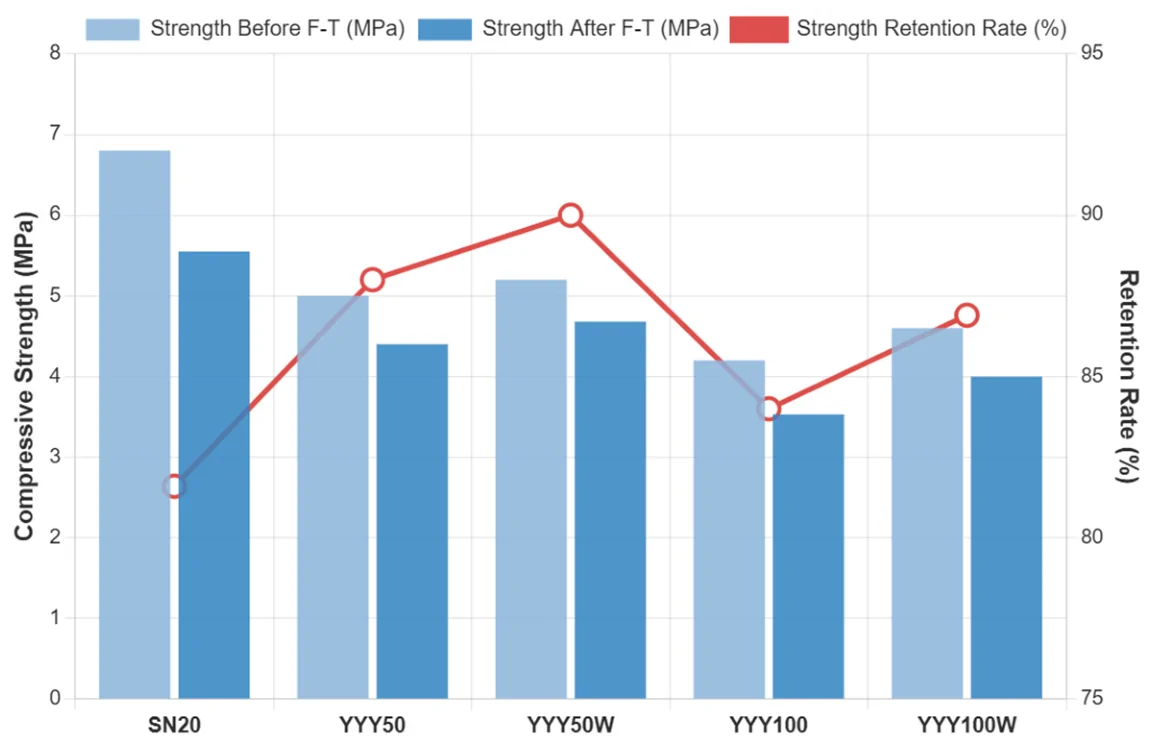
Frost resistance performance after 15 freeze–thaw cycles.

The durability of the AAC specimens was evaluated after 15 rigorous freeze–thaw cycles. The compressive strength retention and structural integrity results are presented in Figure 3.

After 15 cycles, all specimens remained structurally intact without visible macroscopic spalling or cracking, and the mass loss for all groups was tightly controlled within 0~3 g. Notably, the inclusion of oil shale residue significantly improved the frost resistance in terms of strength retention. The baseline SN20 exhibited an 81.6% strength retention rate. In contrast, the optimized YYY50W group exhibited superior structural integrity, with a strength retention rate (R) of 90.0%.

Notably, the inclusion of oil shale residue significantly reversed its initially “negative” effect when subjected to freeze–thaw weathering. The baseline SN20 exhibited an 81.6% strength retention rate. In striking contrast, the optimized YYY50W group demonstrated superior long-term stability, achieving a strength retention rate (R) of 90.0%. This enhancement is fundamentally driven by the microstructural buffering capacity [3,24]. Although the absolute initial strength of SN20 is higher, its interconnected pores are more susceptible to water ingress [25]. Conversely, the YYY50W matrix features abundant, disconnected spherical pores (78.6% closed-pore ratio) that act as effective expansion chambers, safely accommodating the hydrostatic pressure generated by internal ice formation [26].

This durability enhancement represents a significant advancement over traditional formulations. According to recent comprehensive evaluations, standard fly-ash-based AAC typically struggles to maintain a strength retention rate above 85% after 15 freeze–thaw cycles, often suffering from severe microcrack propagation due to rapid capillary water absorption [4,15]. Therefore, the outstanding 90.0% retention rate achieved by the optimized YYY50W matrix not only mitigates the inherent vulnerabilities of pure fly ash systems but also sets a highly competitive benchmark for solid-waste-based AAC in extreme cold climates.

### 3.4. Microstructural Evolution (XRD and SEM)

**Figure 4:**
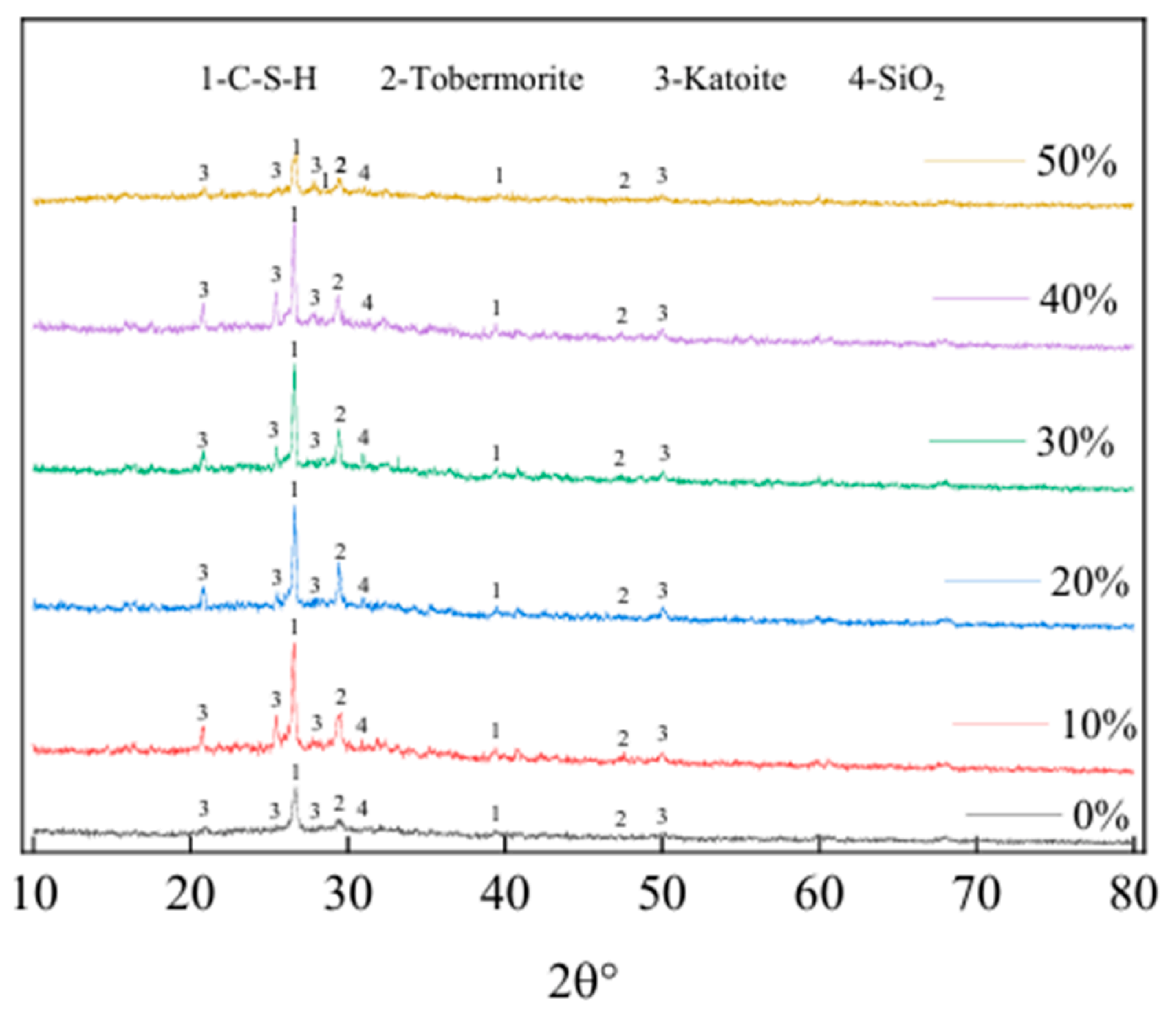
XRD patterns of AAC specimens with different oil shale residue contents.

The mineralogical evolution was tracked via XRD (Figure 4). It is important to note that while preliminary phase screening was conducted across a gradient of replacements (0% to 50%), the macroscopic evaluation (Section 3.2) focused specifically on the critical structural thresholds (baseline and optimal 50% replacement). The XRD analysis revealed that the primary hydrothermal products in all AAC specimens were highly crystalline Tobermorite, C-S-H gels, and Katoite. As the oil shale residue replacement increased to 50%, the diffraction peaks corresponding to Tobermorite broadened and slightly weakened compared to the 0% baseline. This indicates that while active SiO_2_ and Al_2_O_3_ in the residue participate in the CaO-SiO_2_-H_2_O reaction, the resulting tobermorite exhibits slightly lower crystallinity.

**Figure 5:**
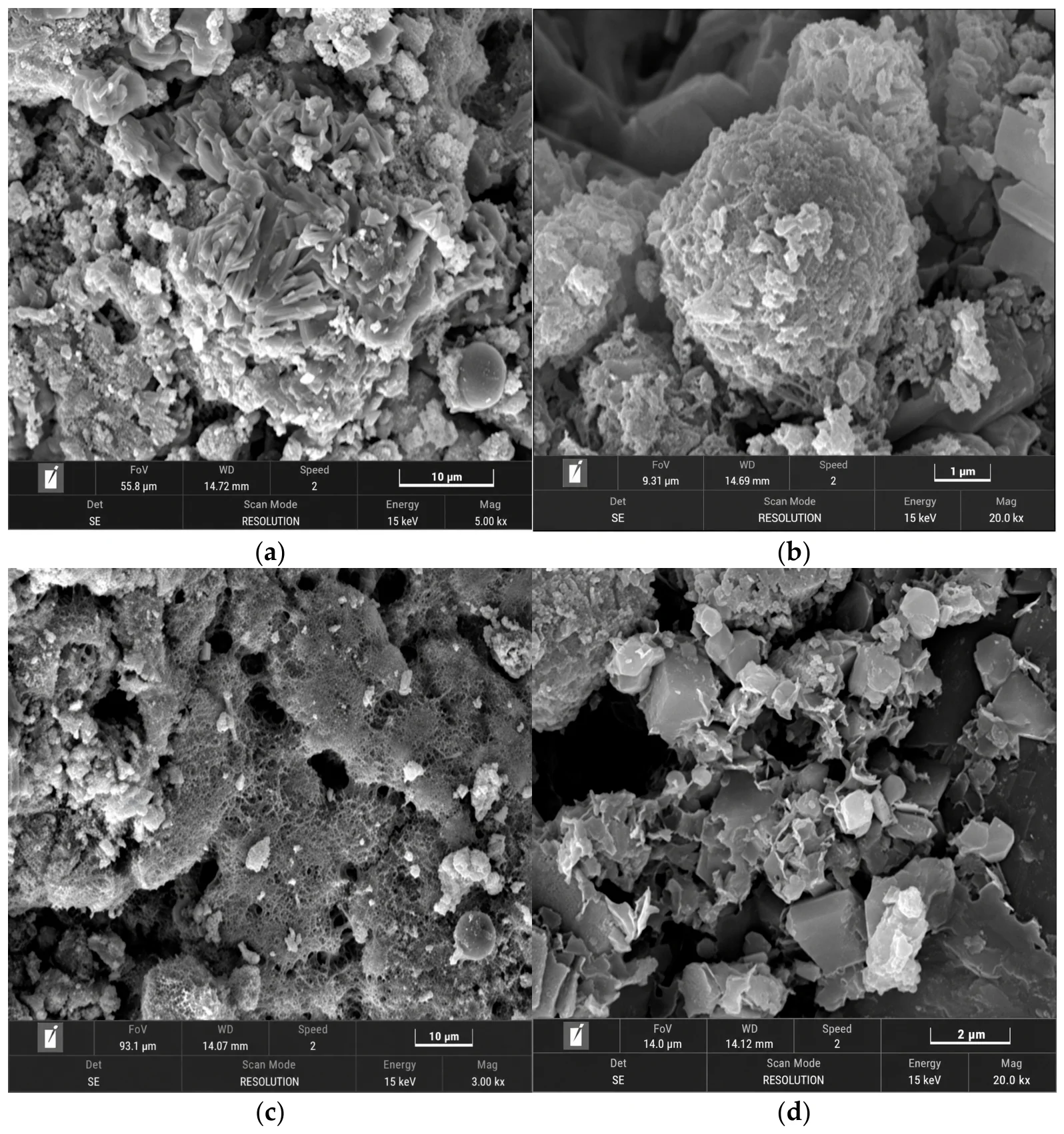
SEM morphology of the AAC matrices: (**a**) overview of SN20 matrix at 10 μm scale; (**b**) high-magnification detail of SN20 showing spherical hydration aggregates at 1 μm scale; (**c**) overview of YYY50W matrix at 10 μm scale; (**d**) high-magnification detail of YYY50W showing dense blocky/plate-like hydration clusters at 2 μm scale.

To further elucidate the macroscopic behavior, SEM observations (Figure 5) were conducted to verify the micro-morphology of the hydration products. The scales were carefully examined to ensure accurate interpretation. In the baseline SN20 group (Figure 5a,b), the matrix overview (10 μm scale) displays a highly porous interconnected skeleton, while the high-magnification image (1 μm scale) reveals that the hydration products predominantly consist of spherical clusters enveloped by amorphous C-S-H gels.

To further elucidate the macroscopic behavior, SEM observations (Figure 5) were conducted to verify the micro-morphology of the hydration products. The scales were carefully examined to ensure accurate interpretation. In the baseline SN20 group (Figure 5a,b), the matrix overview (10 μm scale) displays a highly porous interconnected skeleton, while the high-magnification image (1 μm scale) reveals that the hydration products predominantly consist of spherical clusters enveloped by amorphous C-S-H gels. In contrast, the optimized YYY50W group exhibits a distinctly different morphology. The 10 μm overview (Figure 5c) demonstrates a much denser and refined local matrix skeleton, which corroborates the high closed-pore ratio observed in the MIP tests. At higher magnification (Figure 5d, 2 μm scale), the hydration products in YYY50W manifest as large, tightly packed blocky and plate-like tobermorite intergrowths. While these massive, dense clusters restrict capillary water penetration (thereby significantly improving freeze–thaw durability by starving the matrix of freezable water), they possess fewer interlocking contact points compared to the diffuse gels seen in SN20. This microstructural shift perfectly elucidates why the initial compressive strength slightly decreased, yet the long-term frost resistance retention rate improved so dramatically.

## 4. Conclusions

In this study, oil shale residue was utilized as a high-volume replacement for fly ash to prepare autoclaved aerated concrete (AAC). The fresh properties, mechanical performance, thermal insulation, and microstructural frost resistance mechanisms were systematically evaluated. The main conclusions are summarized as follows:

Directly substituting pure fly ash with oil shale residue slightly reduces the initial physical properties and slurry flowability. However, adjusting the water-to-binder ratio to 0.66 mitigates these issues, ensuring uniform gas expansion. Despite this minor performance trade-off, the optimized mixture with a 50% replacement ratio (YYY50W) exhibits a reliable compressive strength of 5.2 MPa and a thermal conductivity of 0.1654 W/(m·K). This fully satisfies standard structural and insulation requirements while facilitating the large-scale recycling of solid waste.

Furthermore, the incorporation of oil shale residue significantly enhances the long-term frost durability of the AAC matrix. Microstructural evaluations via XRD and MIP confirm that the residue promotes the formation of low-crystallinity tobermorite and abundant C-S-H gels. These hydration products effectively seal internal micro-voids, increasing the closed-pore ratio to 78.6%. Serving as a hydrostatic expansion buffer, this refined pore network enabled the YYY50W mixture to maintain a 90.0% strength retention rate and its structural integrity after 15 freeze–thaw cycles, substantially outperforming the baseline fly ash group (81.6%).

Overall, these findings provide a practical reference for utilizing oil shale residue in cold-region building materials. The refined closed-pore network of this optimized AAC matrix establishes a robust structural foundation for the future integration of phase-change materials (PCMs) to achieve combined durability and active thermal regulation.

## Figures and Tables

**Table 1:** Chemical composition of main raw materials (wt.%).

Material	SiO_2_	Al_2_O_3_	Fe_2_O_3_	CaO	MgO	SO_3_	Loss on Ignition
Oil Shale Residue	55.84	18.59	4.78	10.70	-	1.89	-
Fly Ash	54.50	19.20	4.90	4.50	2.00	0.60	14.30
Cement	21.70	4.76	3.57	63.01	-	1.89	2.14

**Table 2:** Mix proportions of AAC specimens (kg/m^3^).

Group ID	Fly Ash (Siliceous)	Oil Shale Residue (Siliceous)	Cement (Calcareous)	Lime (Calcareous)	Gypsum (Calcareous)	Al Powder	W/B Ratio
SN20	1072	0	320	160	48	1.44	0.60
YYY50	536	536	320	160	48	1.44	0.60
YYY50W	536	536	320	160	48	1.52	0.66
YYY100	0	1072	320	160	48	1.52	0.60
YYY100W	0	1072	320	160	48	1.52	0.66

**Table 3:** Pore structure parameters of selected AAC specimens.

Group ID	Total Porosity (%)	Average Pore Diameter (μm)	Closed-Pore Ratio (%)
SN20 (Baseline)	71.5	160.2	62.4
YYY50 (Un-optimized)	74.8	105.4	55.1
YYY50W (Optimized)	72.3	95.8	78.6

## Data Availability

The original contributions presented in this study are included in the article. Further inquiries can be directed to the corresponding author.
